# Supplementary material for: Damage-tolerant material design motif derived from asymmetrical rotation
Source: Nat Commun. 2022 Mar 11;13:1289. doi: 10.1038/s41467-022-28991-5 (PMC8917193; doi:10.1038/s41467-022-28991-5)
Supplement: Supplementary file 1 — Supplementary Information [file 41467_2022_28991_MOESM1_ESM.pdf]

Supplementary Information

Damage-tolerant material design motif derived from asymmetrical rotation

Wei Wang[1,2†], Shu Jian Chen[2*†], Weiqiang Chen[3], Wenhui Duan[1*], Jia Zie Lai[1] and Kwesi Sagoe-Crentsil[1]

[1]Department of Civil Engineering, Monash University, Clayton, Victoria 3800, Australia

[2]School of Civil Engineering, The University of Queensland, St Lucia, Queensland 4072, Australia

[3]Department of Mechanical, Aerospace and Civil Engineering, School of Engineering, The University of Manchester, Manchester, M13 9PL, United Kingdom

* Corresponding authors: shujian.chen@uq.edu.au (S.J. Chen); wenhui.duan@monash.edu (W.H. Duan).

† These authors contributed equally to this work.

This Supplementary Information includes Supplementary Figures, Supplementary Tables and Supplementary Notes.

Supplementary Figures

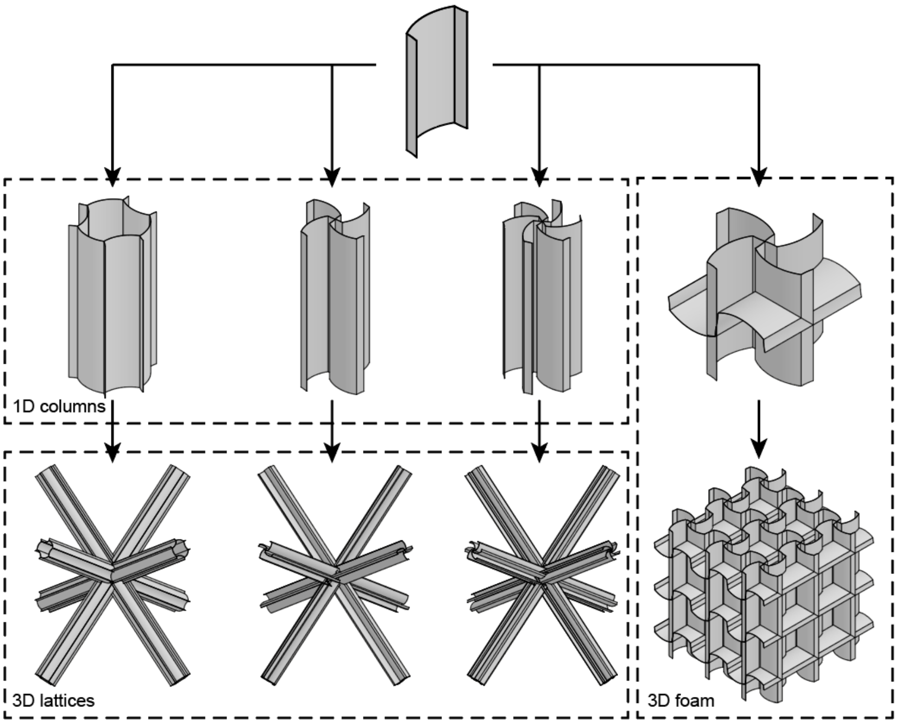

Supplementary Fig. 1. Assembling examples using segmented U-shape units. Single U-shape units can be assembled into 1D columns, 2D honeycombs (Fig. 2a in main text), 3D lattices and 3D foam structures.

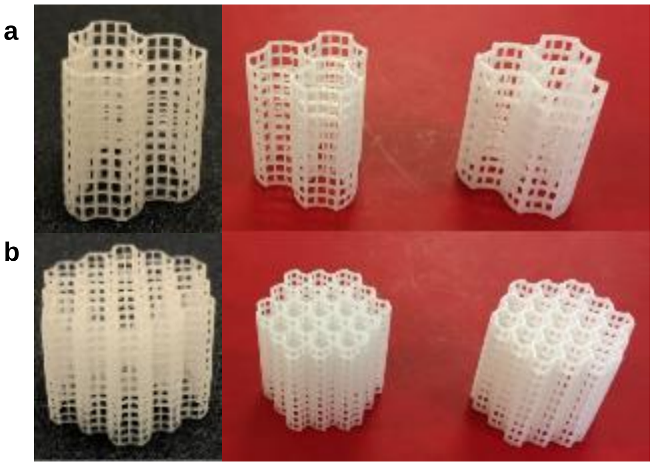

Supplementary Fig. 2. Images of printed polymer scaffolds. **a** SH2 structural configuration with different element sizes. Average weight of scaffold is 0.32, 0.62 and 0.83 g, respectively (left to right). **b** SH1 structural configuration with different element sizes.

Average weight of scaffold is 0.69, 1.32 and 1.94 g, respectively (left to right). Fine elements with overly small size failed to maintain the designed structure geometry. SH, segmented honeycomb

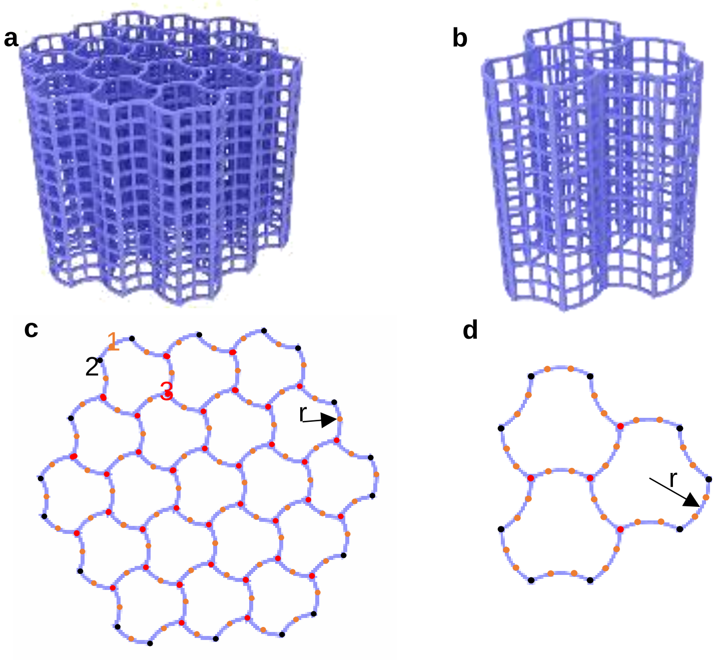

Supplementary Fig. 3. Schematic of the polymer scaffolds. a Segmented honeycomb structure (SH2) and **b** Segmented honeycomb structure (SH1). **c**, **d** Top view of tubular structures with labelled column type 1 (orange), type 2 (black) and type 3 (red).

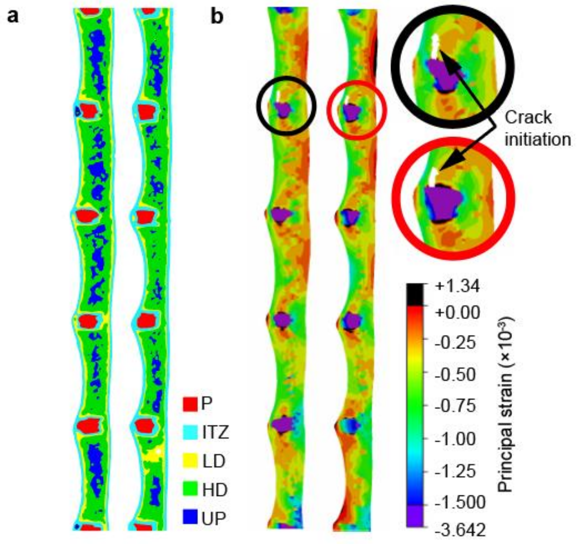

Supplementary Fig. 4. Extended finite element method (XFEM) modelling of segmented honeycomb. a Typical vertical cross-sections showing the different phases within a segmented honeycomb, which are polymer (P), interfacial transition zone (ITZ), low-density hydration products (LD), high-density hydration products (HD) and unhydrated cement

particles (UP). Details shown in Supplementary Note 7. **b** Results from XFEM simulation illustrating the strain distribution of segmented honeycomb when subjected to load from the top. Strain values are presented in the colour bar. Magnified view of circled regions shows crack initiation in the segmented honeycomb and formation of tensile regions (positive strain) around the polymer scaffold.

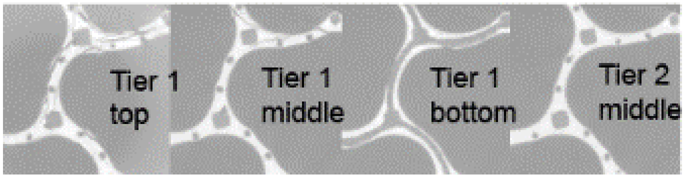

Supplementary Fig. 5. Typical horizontal cross-sections at loading state S2 (Fig. 3b**).** Images obtained by X-ray micro-CT scan

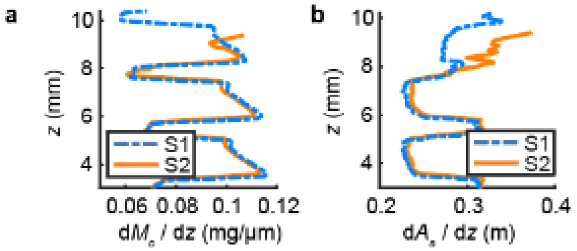

Supplementary Fig. 6. X-ray micro-computed tomography (micro-CT) scan results. Graphs comparing (**a**) mass of hardened cement paste (HCP), dM_c/dz and (**b**) surface area of the HCP, dA_s/dz between loading state S1 (Fig. 3a) and S2 (Fig. 3b)

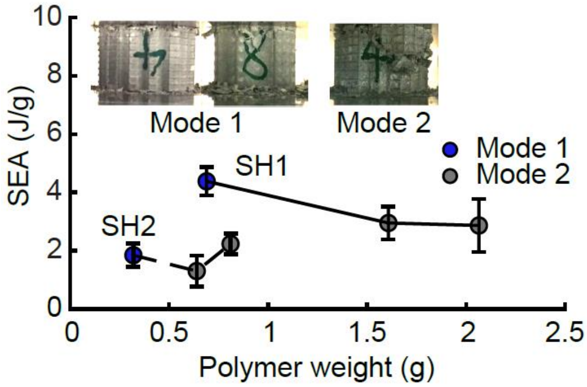

Supplementary Fig. 7. Changes in failure mode and specific energy absorption with the increase in polymer weight. Details of polymer weight and element sizes are presented in Supplementary Table 2. Mode 1 represents regular periodical progressive failure and Mode 2 represents irregular non-periodical progress failure. The error bars indicate the standard deviatiation.

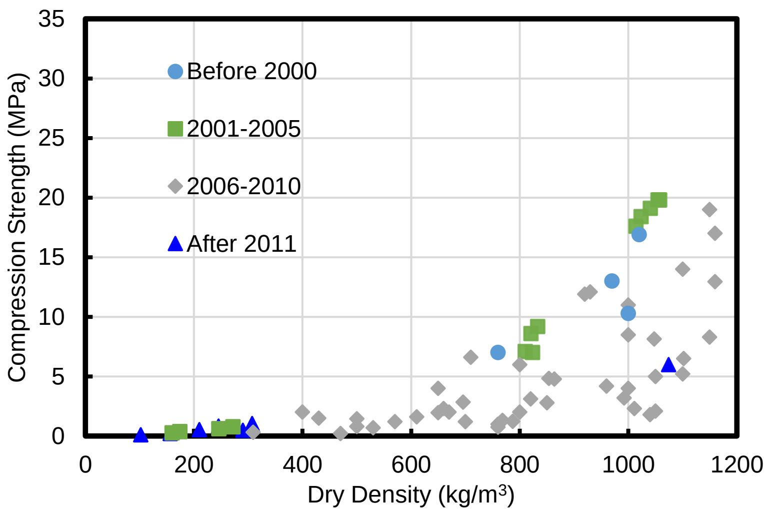

Supplementary Fig. 8. Literature review of lightweight cementitious material. Graph showing compressive strength of foam concrete from research conducted before year 2000 [1-3], from year 2001 to 2005 [4-8], from year 2006 to 2010 [9-17] and after 2011 [18-20].

Supplementary Tables

Supplementary Table 1. Comparison of fabrication techniques in the literature for bioinspired materials [21]

Method	Applications	Limitations
Biomimetic mineralisation	● Nacre−like composites (layered structure)<br>● Can be used to grow particles, capsules and films<br>● Mineralisation of an organic scaffold to create a practical 3D structure	● Low efficiency<br>● Limit of sample size: only able to produce micrometre-sized specimens<br>● Mechanical properties that fall short of those of their natural counterparts
Freeze-casting	● Used for cellular structures and nacre-like structures	● Need to have specifically designed equipment<br>● Limit of sample size: lamellar orientation larger than centimetre will be difficult<br>● Cannot replicate all the nuances of the natural counterpart
Additive manufacture	● Powerful platform that is able to produce various structures (include layered structure, cellular structure, helical structure, etc) compared with others with minimum feature ranges from tens of micrometres to one sub-micrometre	● Limited materials available<br>● Limit of sample size: sample is usually small for high-resolution printing<br>● Bioinspired materials are usually hybrids that combine dissimilar materials, which makes things more complicated

Supplementary Table 2. Sizes of polymer scaffold elements of segmented honeycomb (SH) structures shown in **Supplementary Fig. 3**.

**Design type**	**Column size (mm)**			**Beam size (mm)**	**Cell size, r (mm)**	**Weight (g)**	**Strength ratio (%)**
	**Type 1**	**Type 2**	**Type 3**				
SH1	0.31×0.31	0.44×0.44	0.61×0.61	0.31×0.35	2.9	0.32	1.6
	0.44×0.44	0.62×0.62	0.88×0.88	0.31×0.7	2.9	0.62	14.8
	0.53×0.53	0.76×0.76	1.07×1.07	0.31×0.93	2.9	0.83	21.1

SH2	0.33×0.33	0.55×0.55	0.77×0.77	0.33×0.3	5.5	0.69	1.3
	0.46×0.46	0.78×0.78	1.1×1.1	0.33×0.7	5.5	1.32	5.3
	0.57×0.57	0.96×0.96	1.34×1.34	0.33×0.93	5.5	1.96	10.8

The strength ratios were calculated based on the compressive strength of the polymer scaffold over the compressive strength of coated segmented honeycombs.

Supplementary Table 3. Numerical results of all tested structures

**Design type**	**Poly., g**	**Air, vol %**	**Solid, vol%**	**Solid, vol%**		**Solid, wt%**		**Density, kg/m^3**	**Compr. strength, MPa**	**Ave. spec. compr. strength, MPa·kg·m^{-3}**
				**Poly.**	**Cem.**	**Poly.**	**Cem.**			
SH1	0.69	73.3	26.7	20.6	79.4	14.7	85.3	448±57	7.2±1.5	0.0160
	1.61	70.0	30.0	42.3	57.7	32.6	67.4	403±4	5.6±0.1	0.0139
	2.06	65.9	34.1	47.8	52.2	37.7	62.3	439±23	7.1±0.4	0.0161
SH2	0.32	84.4	15.6	22.0	78.0	15.7	84.3	259±7	3.8±0.5	0.0147
	0.64	79.2	20.8	32.9	67.1	24.5	75.5	293±6	3.2±0.2	0.0110
	0.78	76.5	23.5	36.1	63.9	27.2	72.8	316±43	4.4±1.0	0.0138

The volume fractions relate to the respective bulk volume that includes air and solid. The solid includes the polymer scaffold (Poly.) and cement (Cem.). The average density and compressive (Compr.) strength are calculated based on at least four measurements. The average specific compressive strength (Ave. spec. compr. strength) is calculated using the average compressive strength divided by the average density. SH, segmented honeycomb

Supplementary Table 4. Properties of coating materials [22,23]

		No.	Material	Young's Modulus (GPa)	Compressive Strength (MPa)
Coatings	Cementitious	1	Cement	30	108
		2	PMMA bone cement	2	116
		3	Calcium phosphate cements (CPC): brushite	24.3	55
		4	Calcium phosphate cements (CPC): apatite	13.5	12
		5	Calcium phosphate cements (CPC): monetite	7.1	35
		6	Zinc Phosphate cement	13.2	117
	Ceramics	7	Sapphire	400	2000
		8	Cermet	440	3650
		9	Silicon Carbide	440	972.4
		10	Alumina	360	3095
		11	Aluminium Nitride	325	2335
		12	Silicon Nitride	231.5	3012
		13	Mullite	155.5	935
		14	Zirconia	142.15	3200
		15	Forsterite	150	586
		16	Cordierite	70	350
		17	Steatite	103	568.5
		18	Boron Carbide	417	4135
		19	Boron Nitride	59.75	382.5
		20	Graphite	16.9	415
		21	Titanium Diboride	420	3561.95
		22	Tungsten Carbide	643	5090
		23	Fused silica	73	1600
		24	Silicon	160	3330
	Glasses	25	Borosilicate	73.5	2000
		26	Soda Lime	71	330
		27	Pyroceram	120	850

Supplementary Table 5. Properties of scaffold materials [24,25]

		No.	Material	Young's Modulus (GPa)	Compressive Strength (MPa)
Scaffold	Polymer	28	FullCure (FC) 720	2.87	84.3
		29	ABS	2.5	65
		30	Acrylic	3.1	124
		31	PLA	3.5	55.85
		32	ASA	2.2	50
		33	PET	2.95	80
		34	PETG	2.1	55
		35	PC	1.95	140
		36	PEEK	3.855	124
		37	PEKK	3.35	162.5
		38	PEI	2.96	151
		39	PP	1.344	48
		40	Nylon 6/6	2.93	86.2
		41	LDPE	0.35	9.65
		42	HDPE	0.85	31.7
		43	PTFE	1.3	23.5
	Metalic	44	Stainless Steel 316	197.5	240
		45	Stainless Steel 420	200	1480
		46	Copper	127	187.5
		47	Bronzes	95	382.5
		48	Brass	117	165
		49	Inconel 625	207	500
		50	Inconel 718	200	1150
		51	Aluminum (AlSi10Mg)	70	245
		52	Titanium	102.5	150
		53	Ti6Al4V	114	964
		54	Cobalt	207	610
		55	Silver	71.5	172.5
		56	Platinum	163	110
		57	Gold	98	150

Supplementary Table 6. Chemical composition of general-purpose ordinary portland cement powder

Al_2O_3	SiO_2	CaO	Fe_2O_3	K_2O	MgO	Na_2O	SO_3	LOI
4.7	19.9	63.9	3.4	0.5	1.3	0.2	2.6	3.0

Supplementary Notes

Supplementary Note 1. Comparison between segmental and overlapping structures

Both segmental and overlapping structures are consisted of multiple individual elements (such as segments, plates or scales). Here, a detailed comparison is given to distinguish the segmental structure from the overlapping structure.

First of all, these two structures are designed for different loading conditions and serving for different functions. As shown in Supplementary Fig. 9a, the segmental structure is usually loaded and exhibits a superior compressive strength in its longitudinal direction. Hence, the segmental structure can be implemented in animals' legs for load bearing purpose (support the self-weight). However, the overlapping structure is not effective to carry load in longitudinal direction. The overlapping structure (such as fish scales) is mainly designed for puncture resistance and the puncture force is in transverse direction [26-29] as shown in Supplementary Fig. 9c. Hence, the overlapping structure is usually used as an armour for protection purposes [26,29-31].

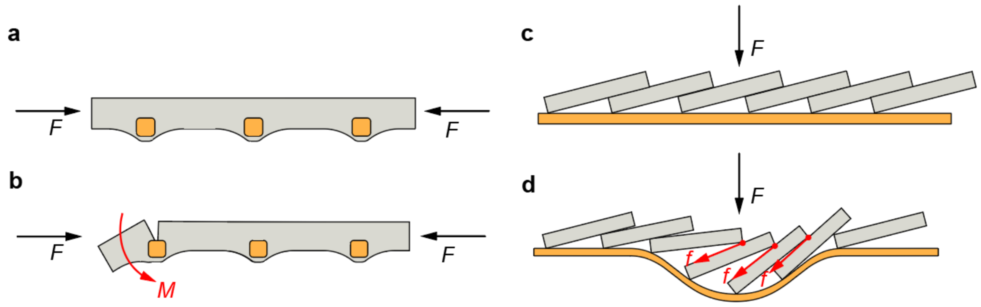

Supplementary Fig. 9. Comparison between segmental structure and overlapping structure. a, b The segmental structure before and after deformation. **c, d** The overlapping structure before and after deformation.

Besides, the energy absorption mechanisms of these two structures are also different. The energy absorption ability of our segmental structure mainly depends on the asymmetrical rotation of the stiff segments around joints (Supplementary Fig. 9b). For example, during the leaping of flea, the segments of flea's leg will rotate asymmetrically around joints to absorb and release energy [32]. However, for the overlapping structure, energy absorption is achieved via the sliding between the individual plates or scales [29,30,33] (Supplementary Fig. 9d). The literature also indicated the individual plates/scales with architectured and curved surfaces increase the friction coefficient between the interface of plates or scales, leading to improved puncture resistance and energy absorption ability [34]. Besides, bending of the individual

plates/scales [30] and brittle failure of the plates/scales [35] under high impact also assist with energy absorption capacity.

Supplementary Note 2. Effect of printing orientation and layer thickness of polymer scaffold

Scanning electron microscopy (SEM). The FEI Magellan™ 400 Field Emission Gun SEM with extreme high resolution was used to observe the micromorphology of the fractured samples after the compression tests. An accelerating voltage and current of 5 keV and 50 pA, respectively, were used.

Four-point bending test. The 4-point bending test was conducted in order to investigate the effect of 3D printing parameters on the performance of 2D plates via a Bose Electroforce 3200 test instrument. The loading rate was set at 0.2 mm/min. A specific design loading head manufactured using the Objet Eden 360 3D polymer printer was used. The total span was 40 mm and the load span was 20 mm. The deformation of the polymer loading head was calibrated using a stainless steel 304 plate with a dimension of 6 × 3 × 0.3 cm.

The printing orientation and layer thickness of the polymer scaffold are important to form the segmental motif. The horizontal layer orientation (H) shows a brittle failure under 4-point bending while the vertical orientations (V1 and V2) showed more ductile failure (Supplementary Fig. 10). The cracks are preferred to propagate along the layer orientations. Hence, when the layer orientations (V1 and V2) were perpendicular to the crack direction (Supplementary Fig. 11), the polymer elements (columns and/or beams) prevented further propagation of cracks and deflected the cracks to the layers' printing orientations (Supplementary Fig. 11). Hence, the structure is able to sustain a larger loading and absorb more energy during the loading process. We also found that sample V1 showed higher loading and energy absorption capacity than V2, resulting from the different surface hydrophilicity of the different surface finishes (Supplementary Fig. 10c–f) where the surface of sample V1 was more hydrophilic and had more fluid deposited. In addition, increasing layer thickness and accelerated printing speed (sample V1 19 μm) decreased the performance of the samples due to weakened layer interface bonding (Supplementary Fig. 10g, h). It needs to be noted that in the 3D scaffolds, vertical orientations, V1 and V2, always exist simultaneously. Hence, in order to minimise spatial variation and ensure joint rotation, the horizontal printing orientation was used instead of the vertical.

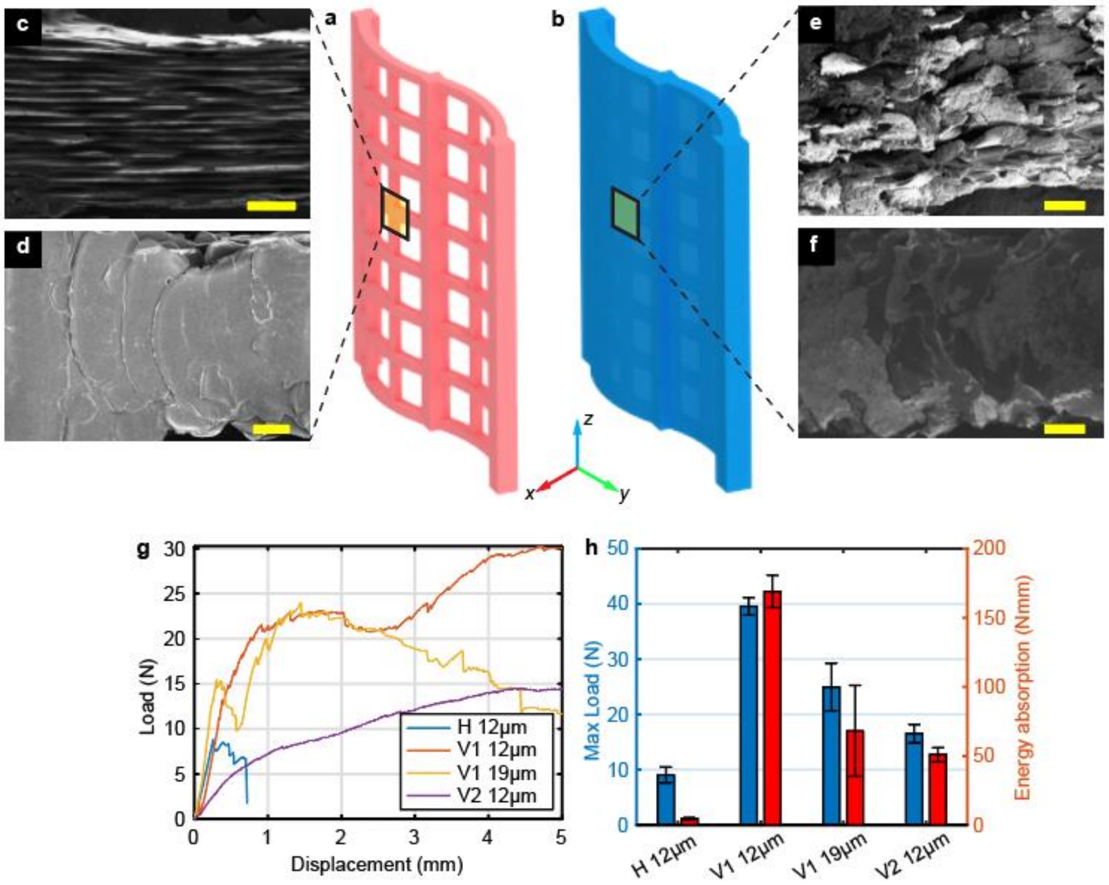

Supplementary Fig. 10. Effect of 3D printing parameters on polymer scaffold. a, b Schematic of the 2D plate before and after cement coating. **c, d** SEM images showing the different surface finishes of the printed polymer scaffolds through controlling of the printing parameters: (**c**) uneven surface showing the layered structure for printing with horizontal orientation (H, printing in *xy* plane) or vertical orientation 1 (V1, printing in *xz* plane) and (**d**) smooth surface finish for printing with vertical orientation 2 (V2, printing in *yz* plane). Scale bars = 100 µm. **e, f** SEM images showing the surfaces of the polymer scaffold after the hierarchical 2D cementitious plate was loaded under 4-point bending: (**e**) uneven surface shown in (**c**) and (**f**) smooth surface shown in (**d**). Scale bars = 100 µm. **g**, Typical loading curves of different batches of 2D cementitious plate under 4-point bending. **h**, Comparison of maximum load (left y-axis) and energy absorption abilities (right y-axis) of different batches of 2D cementitious plate under 4-point bending. SEM, scanning electron microscopy

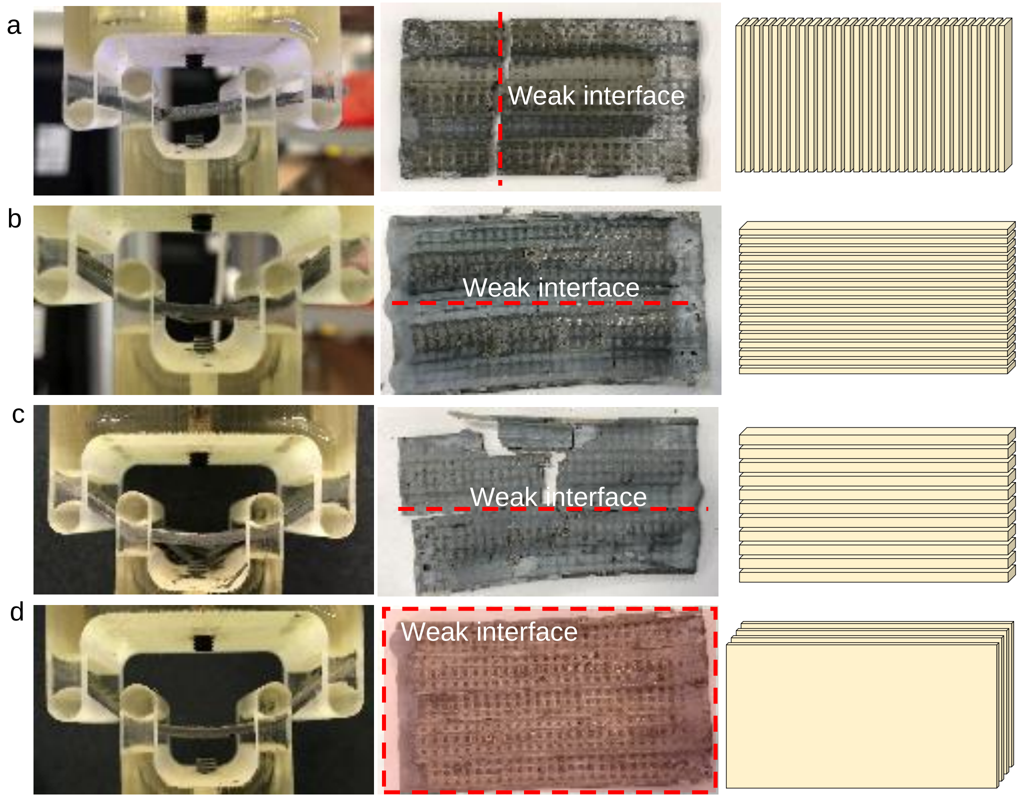

Supplementary Fig. 11. Images of 2D cementitious plates loaded with 4-point bending. a H 12 µm; **b** V1 12 µm; **c** V1 19 µm; and **d** V2 12 µm. The red dashed lines and the square regions indicate the orientation of the weak interface due to 3D printing. From left to right are images of 2D plates during and after loading and a schematic of the printing thicknesses and orientations of the scaffold. The thickness of the printing layer is not in scale.

Supplementary Note 3. Lattice-Boltzmann method (LBM) simulation

LBM was used to simulate the interaction between the cementitious material and polymer scaffold. In this simulation, fluid that had properties similar to those of fresh cement paste was allowed to drip onto the scaffold to observe the behaviour of the fluid.

LBM algorithm and models. The LBM simulation was carried out using open source software Palabos as the solver. The LBM is essentially a discrete-velocity Boltzmann equation [36,37], which can be expressed as:

$$f_\alpha^\sigma(\mathbf{x}+\mathbf{e}_\alpha\delta_t, t+\delta_t) = f_\alpha^\sigma(\mathbf{x},t) - \frac{1}{\tau^\sigma}\left[(\mathbf{x},t) - f_\alpha^{\sigma(eq)}(\mathbf{x},t)\right] \quad (1)$$

where $f_\alpha^\sigma(\mathbf{x},t)$ is the particle distribution function (PDF) of the fluid component σ, at given position $\mathbf{x}$ and time t; α ranges from 0 to 18 for a 3D 19-velocity lattice (D3Q19) model; δ_t is the time step whereas $\mathbf{e}_\alpha$ denotes the discrete lattice velocity; $f_\alpha^{\sigma(eq)}(\mathbf{x},t)$ denotes the local equilibrium PDF; and τ^σ is the relaxation time.

From equation (1), the local PDF of the fluid component can be expressed as:

$$f_\alpha^{\sigma(eq)} = w_\alpha\rho_\sigma\left[1 + \frac{\mathbf{e}_\alpha\cdot u_\sigma^{eq}}{c_s^2} + \frac{\left(\mathbf{e}_\alpha\cdot u_\sigma^{eq}\right)^2}{2c_s^4} - \frac{u_\sigma^{eq}\cdot u_\sigma^{eq}}{2c_s^2}\right] \quad (2)$$

where w_α is weighting factor depending on the lattice model, ρ_σ is the macroscopic density, c_s is the lattice speed of sound equal to $1/\sqrt{3}$, and $u_\sigma^{eq}$is the macroscopic velocity.

To handle the large viscosity ratio associated with simulating a liquid surrounded by air, a free-surface model, with a lattice discretisation of 3D 19-velocity (D3Q19) was used. As cementitious materials are non-Newtonian, the Carreau model was adopted in this simulation. The dynamic viscosity μ of fluid in this model can be expressed as [38]:

$$\mu = \mu_\infty + (\mu_0 - \mu_\infty)(1 + (\lambda \times |S|)^2)^{\frac{(n-1)}{2}} \quad (3)$$

where μ_0 and μ_∞ are viscosities (Pa.s) at zero and infinite strain rates respectively, λ is the relaxation time (s), n is the power index, and $|S|$ is the shear rate. A simplified collision model of the Bhatnagar-Gross-Krook (BGK) model [39] was adopted.

Parameters. μ_0 was set at 40 Pa.s., μ_∞ was varied within the range of 0.005 to 0.282 Pa.s [40,41]. λ and n was varied to fit a specific range values of the yield stress (σ). The shear stress τ, can be calculated using the Newton's law of friction ($\tau = \mu S$).

Other non-rheological parameters include contact angle, surface tension and density. The contact angle and density were set to 0 and 2000 kg/m^3, respectively, according to the properties of the physical specimen. Alternatively, the surface tension (γ) was varied within the range of 0.01 to 0.07 N/m. The combinations of different γ and σ allowed us to study the co-effect of γ and σ on the flow of the fluid.

Initial and boundary conditions. In this simulation, periodic boundary condition was adopted in the horizontal x- and y-axes. In the z-axis, two tiers of the segmented honeycomb structure were simulated so excess fluid was allowed to accumulate at the bottom instead of re-entering the simulation box from the top. In the initial stage of the LBM simulation, a set volume of cementitious fluid was initiated on top of the structure and allowed to flow downwards until reaching equilibrium.

Stopping Criteria. The simulation was allowed to run until equilibrium was achieved. The equilibrium criteria were taken to be when the magnitude of the strain rate was was less than 2.7×10^{-12} kg·m/s.

Supplementary Note 4. Gibbs free energy change during film formation

In order to understand the cement film formation process and the geometry of the cement, the Gibbs free energy changes during the film formation process were calculated. To simplify the calculation process, we made the following assumptions: (1) the cross-section of the polymer scaffold is a square; (2) the yield stress of the cement paste assumed to be 0; (2) gravity would not affect the shape of the cement paste; (3) during the flow of the cement paste, the thickness of the cement film would be uniform (Supplementary Fig. 12).

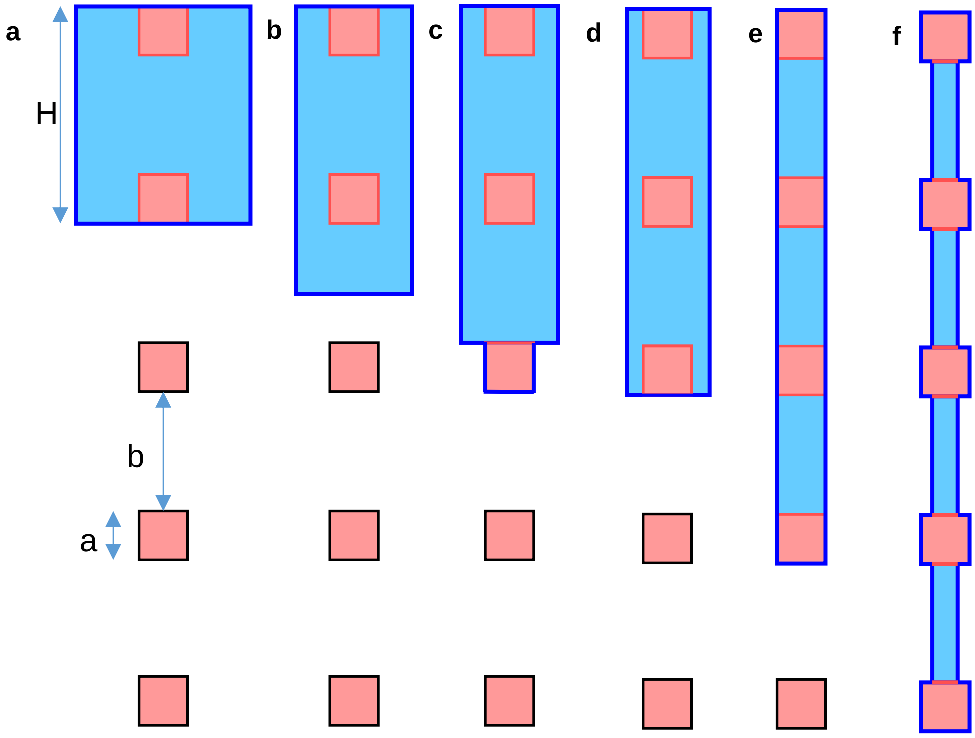

Supplementary Fig. 12. Simplified formation process of cement film on polymer scaffold. **a** A certain volume of cement paste is deposited onto the scaffold. **b** Cement paste flows between the tiers. **c** When the cement paste just touches the third scaffold, it wets the polymer immediately. **d** The cement paste redistributes and covers the third polymer scaffold entirely. **e** The cement paste keeps flow and fills the third void. **f** The cement paste keeps flow and fills the forth void.

As shown in Supplementary Fig. 12, we divided the flow of cement paste into three stages: (1) flow between the tiers (Supplementary Fig. 12b), (2) wetting of the polymer scaffold (Supplementary Fig. 12c) and (3) redistribution of cement paste after wetting (Supplementary Fig. 12d). The polymer scaffold size is denoted as a and the distance between scaffold element is b. A certain volume (V) of cement paste was deposited onto the scaffold to fully cover three

open voids. The total Gibbs energy is the sum of the products of the solid, liquid, and solid–liquid length with their respective surface energies:

$$F = \gamma_S \times 12a + \gamma_{SL} \times 8a + \gamma_L \times 2\left(H + \frac{V + 2a^2}{H}\right) \tag{4}$$

Initially, $H_0 = b + 2a$. Due to gravity acting on the cement paste, the flow is downward (Supplementary Fig. 12b) and the surface energy keeps increasing until wetting of the next tier of the polymer scaffold ($H = 2b + 2a$). The increase in Gibbs free energy during this flowing stage of the second void ΔF_{f2} is:

$$\Delta F_{f2} = \gamma_L \times 2\left(H + \frac{V + 2a^2}{H} - H_0 - \frac{V + 2a^2}{H_0}\right) \tag{5}$$

When the cement paste touches the polymer scaffold, it wets the whole polymer surface immediately (Supplementary Fig. 12c). The Gibbs free energy change is:

$$\Delta F_{w2} = \gamma_{SL} \times 4a + \gamma_L \times 2a - \gamma_S \times 4a \tag{6}$$

According to Young's equation, the surface energies are related to the contact angle, θ:

$$\gamma_S = \gamma_{SL} + \gamma_L cos\theta \tag{7}$$

Hence, the Gibbs free energy change during wetting becomes:

$$\Delta F_{w2} = 2a\gamma_L - 4a\gamma_L cos\theta \tag{8}$$

Next, the cement paste redistributes to reduce the surface energies and the Gibbs free energy change during the redistribution stage is:

$$\Delta F_{r2} = \gamma_L \times 2\left(\frac{V + 3a^2 - (3a + 2b - H)a}{H} - \frac{V + 2a^2}{2a + 2b}\right) \tag{9}$$

Using the same approach, we can calculate the Gibbs free energy changes for the filling of the third void:

$$\Delta F_{f3} = \gamma_L \times 2\left(H + \frac{V + 3a^2}{H} - 3a - 2b - \frac{V + 3a^2}{3a + 2b}\right) \tag{10}$$

$$\Delta F_{w3} = 2a\gamma_L - 4a\gamma_L cos\theta \tag{11}$$

$$\Delta F_{r3} = \gamma_L \times 2\left(\frac{V + 4a^2 - (4a + 3b - H)a}{H} - \frac{V + 3a^2}{3a + 3b}\right) \quad (12)$$

If we assume $V = 3ab$, it is only able to fully cover three voids and if the cement paste keeps flowing to the fourth void, the thickness of the cement film will be thinner than that of the polymer scaffold (Supplementary Fig. 12f). The Gibbs free energy change during this flow stage is:

$$\Delta F_{f4} = \gamma_L \times \left(2H + \frac{2V}{H - 4a} + 7\left(a - \frac{V}{H - 4a}\right) - 2(4a + 3b + a)\right) \quad (13)$$

The Gibbs free energy change during the wetting is:

$$\Delta F_{w4} = \gamma_{SL} \times 4a + \gamma_L \times \left(2a + 2\left(a - \frac{V}{4b}\right)\right) - \gamma_S \times 4a$$
$$= \gamma_L \times \left(4a - \frac{V}{2b} - 4acos\theta\right) \quad (14)$$

There is no redistribution stage for filling the fourth void. The cumulative change in Gibbs free energy is shown in Supplementary Fig. 13.

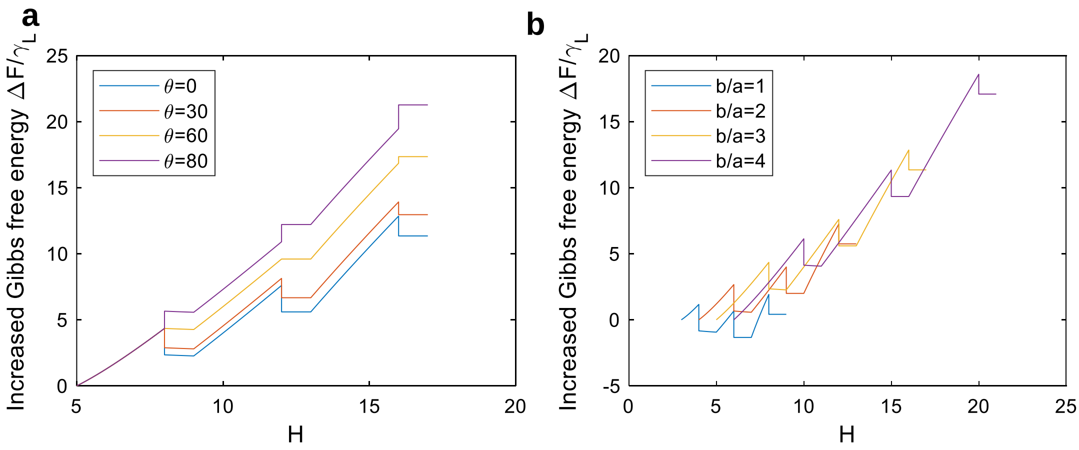

Supplementary Fig. 13. Increased Gibbs free energy during the flowing process of cement paste. a Effect of contact angles on Gibbs free energy change, assuming $V = 3ab$ and $\frac{b}{a} = 3$. **b** Effect of tier height on Gibbs Free energy change, assuming $V = 3ab$ and $\theta = 0$.

Supplementary Note 5. Discrete element method (DEM) simulation

Discrete element method (DEM) simulation was used in our study to investigate the fluid and solid behaviour of the segmented honeycomb. The particle assembly conforming a certain particle-distribution law was firstly generated by our in-house code. Then the fluid flow of the coating material was simulated to investigate the effect of surface tension on the geometry of the coated segmented honeycomb. Finally the particle assembly was going through compression test as is in the experimental part. For the fluid behaviour simulation, the LAMMPS was used and for the solid one, the PFC 3D was used.

Modified JKR model. Note that for the fluid flow simulations of coating materials, only normal contact forces between particles are computed, and the tangential contact forces and moment are assumed to be zero during the simulation. In this way the system can fastly achieve a steady relaxed state. Therefore, a modified Johnson-Kendall-Roberts (JKR) model (Supplementary Fig. 14) describing the normal inter-particle contact force was used in our study to investigate the fluid flow behaviour of the coating materials. The JKR model implemented in the LAMMPS is widely used to simulate the elastic contact with the effect of adhension [42]. However, this model has a very short cohensive distance, meaning that two particles will easily detach in the larger-timestep simulation, causing the particle lost and unstable simulations. Therefore, we modified the original JKR model by introducing a extended cohesive range (Supplementary Fig. 15a). In this way, the neighbouring particles can be effectively attracted, avoiding the escape of the atoms in the relaxation process. Furthermore, we considered the water adsorption on the solid particle surfaces and the thickness of water layer is quantified by k as shown in Supplementary Fig. 14, where the contact between water layers is described by our JKR model with compression component substracted and the one between solid particles is quantifield by Hertz contact model.

Parameters setup in modified JKR model. The parameters below is adopted in our simulations. The parameters are self-explained by Supplementary Fig. 14. The surface tension (γ) we adopted here was 0.072 J/m2 which is the surface tension of water. The Possion's ratios (υ_1, υ_2) were 0.3. E_1 was 26.5 MPa, which was determined by matching the particle interactions with the surface tension. E_2 was 150 MPa, which was determinted by calibrating the mechanical behaviour of the particle interactions with the mechanical performance of cement. We further conducted a sensitivity study on the water thickness, k, as shown in Supplementary Fig. 15b. The thickness adopted in our simulation was k=0.1.

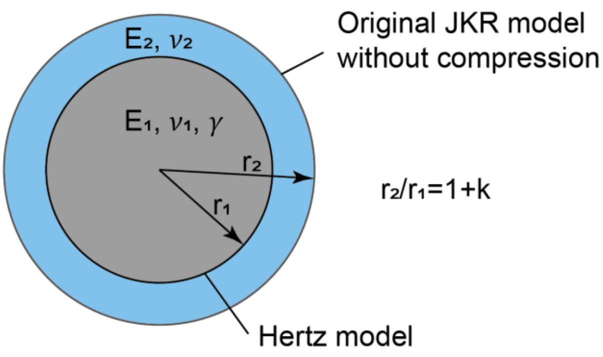

Supplementary Fig. 14. Modified JKR model adopted in our study.

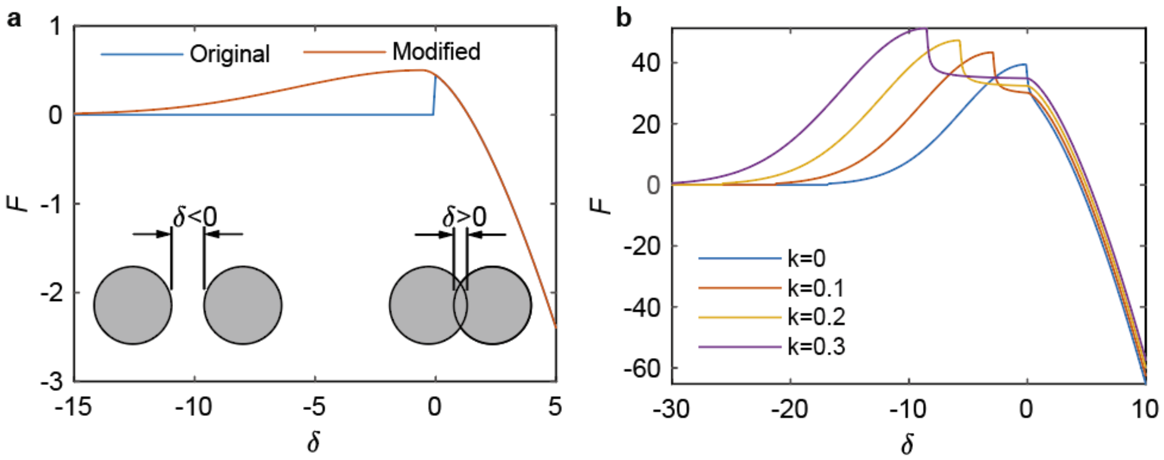

Supplementary Fig. 15. a. the comparision of contact behavior between our modified JKR model and the original JKR model implemented in LAMMPS. **b**. the relation between inter-particle contact behaviours and the water adsorping thickness, k.

NVT ensemble for the relaxation. To better relax the particle system and avoid the escape of very small particle during the relaxation, an in-house code is developed for the implementation of a novel NVT ensemble, where the velocity of every particle is rescaled every 300 timesteps to ensure that the velocity magnitude of every particle can not exceed a specific value (0.1 for our case) and at the same time, the velocity of every particle is set to 0 every 3000 timesteps.

Parallel bonded contact model. The paralleled bonded model developed by Potyondy and Cundall [43] was used in our study to model the solid behaviour (mechanical) of our structure. We follow the symbol convention in Ref [44] and the parameters below were adopted in our simulations, which were found to correctly reproduce the mechanical response of segmented honeycomb under compression.

Supplementary Table 7. Contact parameters used in the PBM model.

Parameter	Values
E_c	11.5 GPa
k_n/k_s	0.95
$\overline{\boldsymbol{k}_n}/\overline{\boldsymbol{k}_s}$	0.95
$\bar{\boldsymbol{\sigma}}_c$	17 MPa
$\bar{\boldsymbol{\tau}}_c$	23 MPa

A Novel Static Loading Scheme. The number of particles in our study reached the scale of ~10^6. And the loading rate in our experiment is 0.1 mm/min with a toal displacement of 10 mm. To reduce the computational cost, we developed a novel static loading scheme to be used in the compression test as below:

Firstly, the strength of every bonds in the particle assembly is kept infinite to ensure that no bond breakage will occur and the overall deforming behaviour of our particle assembly is elastic. The structure was instantly compressed to certain strain by loading platen. After this deformation, the loading platen was kept still.

Secondly, by fixing the loading platen, the whole particle assembly was further relaxed and no bond breakage events occurred during this process.

Thirdly, by fixing the loading platen. The strength of each bonds recovered to their correct value. And the whole particle assembly was relaxed, allowing bond breakage event during this process.

Finally, a new cycle was initiated by applying a new deformation to the particle assembly.

Supplementary Note 6. Multimodal Gaussian distribution fitting for quantitative nanomechanical mapping of Young's modulus distribution curve

The multimodal Gaussian distribution method was also used to fit the Young's modulus distribution curve with several normal distributions curves to represent the different phases in the cement paste. In this case, n=4 in equation (15), which represents the four main phases in the segmented honeycomb, which are (1) polymer/epoxy/defects/voids (P); (2) low-density calcium silicate hydrates (LD); (3) high-density calcium silicate hydrates (HD); (4) unhydrated cement particles (UP). The fitting results are presented in Supplementary Fig. 16 b and the parameters of the fitting curves are summarised in Supplementary Table 8.

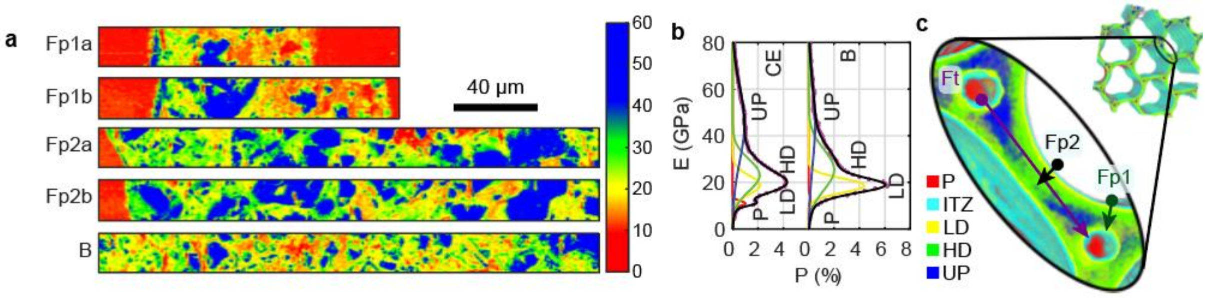

Supplementary Fig. 16 Quantitative nanoscale mechanical (QNM) and X-ray micro-computed tomography scan results. a Elastic modulus (*E*) maps of the hardened segmented honeycomb (SH: Fp1 and Fp2) and hardened bulk cement (B) obtained by QNM characterisation method. **b** Decomposition of *E* into different phases, including polymer scaffold/epoxy/defects/voids (P), low-density C-S-H (LD), high-density C-S-H (HD) and unhydrated cement particles (UP), using multimodal Gaussian distribution curve-fitting. **c** Colour-coded SH based on grayscale values (GSV). Arrows indicate the direction of QNM scans as plotted in (**a**). Scan direction Ft is plotted in **Fig.3**h.

Supplementary Table 8. Coefficient values of multimodal Gaussian curve distribution with 95% confidence interval for segmented honeycomb (SH) and bulk cement (B1) with a w/c ratio of 0.25

	**SH**		**Bulk cement (B)**	
**Coefficient**	**Median**	**Confidence interval**	**Median**	**Confidence interval**
a_1	0.01011	± 0.00122	0.00138	± 0.003141
b_1	11	± 0.14	13	± 3.21

c_1	1.532	± 0.231	2.144	± 4.855
a_2	0.02173	± 0.00506	0.04397	± 0.0031
b_2	18.91	± 0.36	18.62	± 0.2
c_2	5.718	± 0.595	4.346	± 0.341
a_3	0.0213	± 0.00431	0.02	± 0.0076
b_3	23.07	± 1.06	25.41	± 1.13
c_3	9.614	± 0.926	12.15	± 2.82
a_4	0.009295	± 0.000423	0.004949	± 0.002632
b_4	45	± 2.42	44.76	± 22.13
c_4	22.29	± 2.38	24.6	± 16.32

The range of *E* of the primary phases in SH and bulk cement and their volume percentages are summarised in Supplementary Table 9, and were consistent with the values reported in the literature [45].

Supplementary Table 9. Range of *E* of the primary phases and their volume percentages

	LD (GPa)	HD (GPa)	UP (GPa)
SH	18.91 ± 0.36 (23%)	23.07 ± 1.06 (38%)	45 ± 2.42 (38%)
Bulk cement (B)	18.62 ± 0.2 (34%)	25.41 ± 1.13 (44%)	44.76 ± 22.13 (21%)

LD, low-density calcium silicate hydrates; HD, high-density calcium silicate hydrates; SH, segmented honeycomb; UP, unhydrated cement particles

Supplementary Note 7. Decomposition of density data in micro-CT scan into different phases

Dimensionless density data of hardened SH was acquired by reconstructing the images obtained from X-ray micro-computed tomography using Paraview software. The data consisted of the density of every point within the scanned structure, hence a total distribution curve of density data was obtained. As the total distribution curve had several peaks, it was fitted with the multimodal Gaussian distribution method into separate small normal distribution curves that summed to the total curve. The method used for curve-fitting was the non-linear least squares method and the Trust-Region algorithm was adopted. The equation of fitted curve was:

$$P = \sum_{i=1}^{n} a_i * e^{-\left(\frac{x-b_i}{c_i}\right)^2} \tag{15}$$

where n is the total number of distribution curves. Normal distribution curves (n=7) were used in equation (15). The coefficients are presented in Supplementary Table 10.

Supplementary Table 10. Coefficient values of multimodal Gaussian curve distribution with 95% confidence interval

Coefficient	Median	Confidence interval
a_1	4.52×10^{-2}	$\pm 1.07 \times 10^{-3}$
b_1	21110	± 0
c_1	621	± 5.7
a_2	1.14×10^{-2}	$\pm 1.03 \times 10^{-3}$
b_2	21260	± 20
c_2	1144	± 37
a_3	1.79×10^{-3}	$\pm 7.74 \times 10^{-4}$
b_3	24540	± 110
c_3	1696	± 332
a_4	6.48×10^{-4}	$\pm 2.98 \times 10^{-4}$
b_4	29030	± 4810
c_4	3690	± 9900
a_5	8.469×10^{-4}	$\pm 2.755 \times 10^{-3}$
b_5	33020	± 2790

c_5	2054	± 4376
a_6	2.999 × 10^{-3}	± 1.139 × 10^{-3}
b_6	35450	± 1200
c_6	1583	± 400
a_7	2.998 × 10^{-3}	± 2.621 × 10^{-3}
b_7	36290	± 160
c_7	1072	± 215

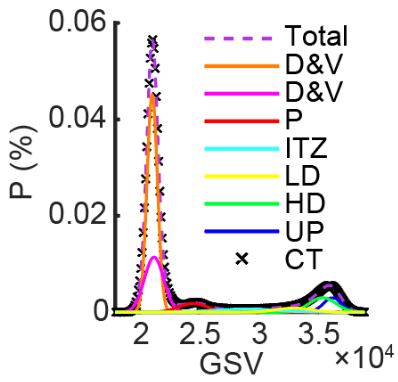

Supplementary Fig. 17. Decomposition of the dimensionless density data via multimodal Gaussian distribution.

The confidence level for the variable intervals above was 95%, and the R-square goodness-of-fit was 0.9998. Each term in the equation represents a normal distribution curve, whereas each curve represents the density distribution of a phase. The 5th, 6th and 7th terms represent the low-density (LD), high-density (HD) and unhydrated cement particles (UP) cementitious phase respectively. The ratio of LD:HD:UP phases was 0.18:0.49:0.33, which conforms well with the ratio determined from QNM mapping of 0.23:0.38:0.38 (Supplementary Table 9).

Each term in equation (15) comprises three variables, with variable *a* controlling the elevation of the curve, variable *b* controlling the median of the curve and variable *c* controlling the deviation of the curve. Because *a* is the controlling variable of the elevation of the curve, it can be tuned within the confidence interval to determine the possibly weakest and strongest cementitious phase compositions. The composition of the cementitious phase was then used to determine the average elastic modulus *E*, as presented in Supplementary Table 11.

Supplementary Table 11. Composition of cementitious phase within the 95% confidence interval range and their corresponding average elastic modulus *E*

	Composition (LD:HD:UP)	Average E (GPa)
Weakest	0.27:0.69:0.04	24.5
Median	0.18:0.49:0.33	30.7
Strongest	0:0.38:0.62	37.4

HD, high-density; LD, low-density; UP, unhydrated cement particles

Supplementary Note 8. Progressive failure material as energy absorption material

Set up of FEM model. A finite element method (FEM) simulation was conducted to investigate the potential application of progressive failure material as an energy absorption material.

The potential application of progressive failure material in a shell structure, which is one of the most widely used and most efficient structures, was investigated. As shown in Supplementary Fig. 18, three types of shell structures were investigated. Each structure contained a thick and stiff element at the bottom, which could be a structural member adopted in airplanes, ships, medical implants such as artificial bones or any other shell structure. This structural member is protected by a lattice structure made from energy absorption material and a protective cover on the top. The details of element sizes are summarised in Supplementary Table 12.

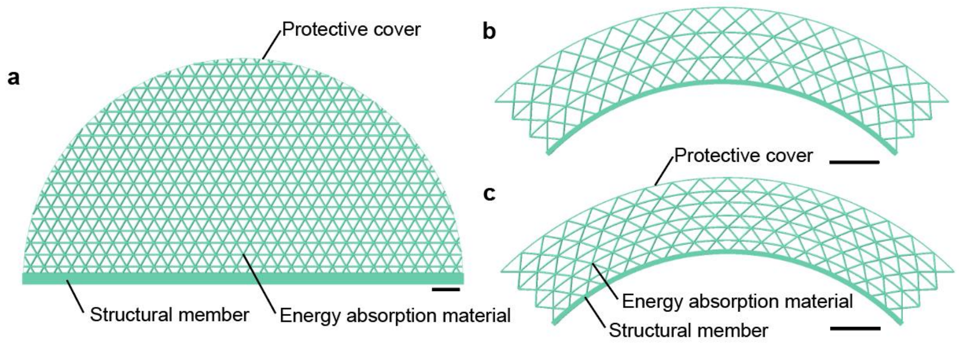

Supplementary Fig. 18. Three types of shell structure, **a** SS1, **b** SS2 and **c** SS3, simulated to investigate the effect of progressive failure material as an energy absorption material compared with ductile materials. Each shell structure contains a thick and stiff structural member at the bottom protected by a lattice structure made from energy absorption material and a protective cover on the top. Scale bar = 1 unit length.

Supplementary Table 12. Cross-section areas of elements in three shell structures (unit length^2)

	Structural member	Energy absorption lattice	Protective cover
SS1	0.16	0.004	0.0001
SS2	0.04	0.004	0.0016
SS3	0.04	0.004	0.0016

Material properties. The structural member and protective cover were made from steel material with a Young's modulus of 200 GPa and yield stress of 281 MPa. Two different types

of energy absorption material were adopted here: progressive failure (PF) material and ductile (Duc) material. To investigate the effect of progressive failure behaviour on energy absorption, the specific energy absorption abilities are controlled to be identical. Hence, the two materials would absorb the same amount of energy when their plastic strains were 0.5 (as shown in **Supplementary Fig. 19**). The plasticity of the PF was set according to the experimental results shown in **Fig. 3**m where multiple peaks were identified during the loading process. The peaks following after the first one were assumed to have 80% of the compressive strength while the valleys between the peaks were assumed to be 20% of the compressive strength. In addition to plasticity, the other properties of the PF and Duc were assumed to be identical.

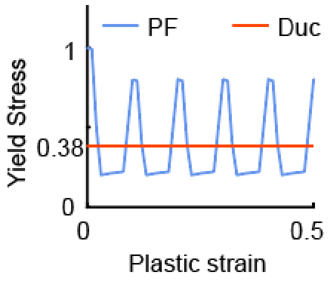

Supplementary Fig. 19. Plasticity of progressive failure (PF) material and ductile (Duc) material adopted in the finite element method (FEM) simulation. The yield strength of the PF is normalised to 1 in the Figure but was set as 70 MPa in FEM model, which is a typical compressive yield strength of cement paste [46].

The effect of damping was also considered in the simulation. The natural frequencies of the systems were calculated first and the Rayleigh damping coefficients were calculated following the method described in the literature [47]. The first and 10th natural frequencies were used for the calculation of damping coefficients. The typical damping ratios of brittle (concrete) and plastic (steel) materials are normally considered as 0.05 and 0.02, respectively [48]. The energy absorption material used here was a PF material, a cementitious material in the experimental demonstration. The damping ratio was assumed to be 0.05. To investigate the effect of the damping ratios, a damping ratio of 0.02 was also simulated and compared with SS3. The damping ratios and damping coefficients are summarised in Supplementary Table 13.

Supplementary Table 13. Damping properties of three shell structures

	ω_1 (Hz)	ω_2 (Hz)	ξ	α	β
SS1	15.45	88.37	0.05	1.31516	0.00096

SS2	25.53	77.29	0.05	1.91884	0.00097
SS3	25.96	78.71	0.05	1.95212	0.00096
SS3	25.96	78.71	0.02	0.78085	0.00038

Energy absorption performance. The energy absorption performance of the PF material was characterised by simulating the shell structure receiving an impact from a rigid body. The mass and impact velocity of the rigid body were controlled and the residual stress within the lattice structure was calculated. The bouncy velocities of the rigid body were also recorded and it was found that >99% of kinematic energy was absorbed during impact. The deformation of the middle of the structural members and the impact locations were also recorded and compared during the impact process. The results are presented in Fig. 3p–q and Supplementary Fig. 20.

The results indicated that compared with ductile materials, the residual stress within the lattice structure was significantly reduced due to the stress-releasing mechanism of the PF material. In addition, deformation in the middle of the structural members and the impact locations were also reduced not only due to the higher strength of the PF material, but also from the PF behaviour, which transfers less stress to the structural member.

Comparing the SS3 with different damping ratios (Supplementary Fig. 20e, f , g, h), the stress-releasing effect was more significant when the damping ratio was higher. The PF material is usually a brittle material, which has a higher damping ratio than the ductile material (such as metals that are usually adopted as an energy absorption material). Hence, the reduction in both residual stress and local deformation of the PF material will be higher compared to ductile material (Duc) if a higher damping ratio is considered.

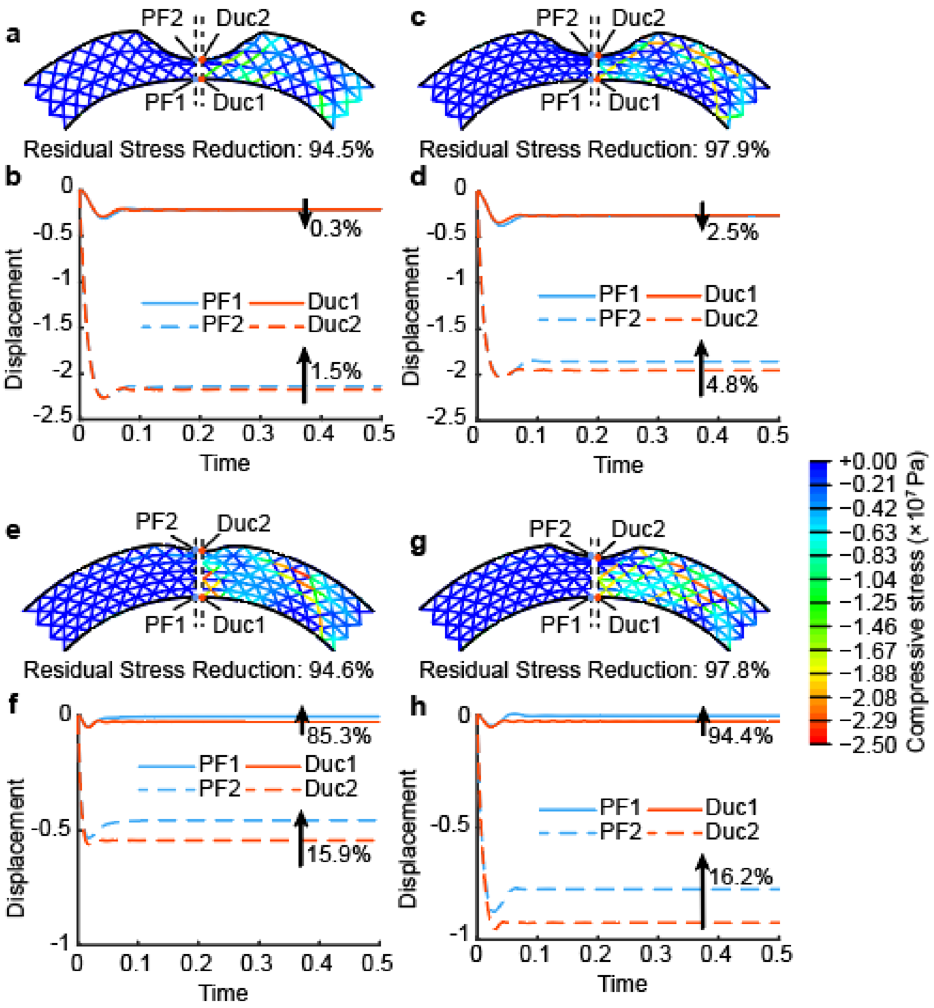

Supplementary Fig. 20. a, b Shell structure SS2 impacted by a rigid body with a mass of 1000 and velocity of 200, damping ratio 0.05. **c, d** Shell structure SS3 impacted by a rigid body with a mass of 1000 and velocity of 200, damping ratio 0.05. **e, f** Shell structure SS3 impacted by a rigid body with a mass of 500 and velocity of 100, damping ratio 0.05. **g, h** Shell structure SS3 impacted by a rigid body with a mass of 500 and velocity of 100, damping ratio 0.02. The residual stress (compressive stress) within the lattice structure is indicated by the colour map.

Supplementary Note 9. Analysis of material utilisation efficiency

Basic concept of extended finite element method (XFEM). The XFEM approach was applied in the ABAQUS program to simulate the initiation and propagation of crack within the structure. The basic concept behind XFEM simulation is to allow formation of cracks within the model by incorporating local enrichment functions into the conventional FEM simulation, in combination with the addition of degrees of freedom (DoF) into the model. The displacement vector u is approximated by the following equation [49]:

$$\boldsymbol{u} = \sum_{I=1}^{N} N_1(x)\left[\boldsymbol{u}_I + H(x)\boldsymbol{a}_I + \sum_{\alpha=1}^{4} F_\alpha(x)\boldsymbol{b}_I^\alpha\right] \tag{16}$$

where u is the approximated displacement vector, N_1 is the shape function associated with a node, u_I is the nodal displacement vectors, $H(x)$ is the jump function of the crack, a_I and b_I^α are the displacement vectors of additional DoF, and $F_\alpha(x)$ is the asymptotic crack-tip function. It should be noted that the first term within the square brackets in equation (16) is associated with all nodes in the model, the second term is associated with enriched nodes around the crack surface and the third term is associated with nodes around the crack tip.

The jump function presented in equation (16) above can be expressed as:

$$H(x) = \begin{cases} 1, & (x - x^*) \cdot n \geq 0 \\ -1, & (x - x^*) \cdot n < 0 \end{cases} \tag{17}$$

where x is a sample point, x^* is the closest point to x on the crack, and n is the unit vector normal to point x^*. Hence, $(x - x^*) \cdot n$ gives the distance from point x to the closest crack.

In our simulation, the cohesive segment approach that uses the traction–separation law was adopted. Because the propagation of a crack can be simulated, the crack-tip asymptotic singularity is not needed and hence, the $F_\alpha(x)$ term is eliminated from the displacement vector approximation equation.

Setup of XFEM simulation. A cross-section of segmented honeycomb obtained from X-ray micro-computed tomography was used to create the distribution of materials as shown in Supplementary Fig. 21a. A model of a 2D planar deformable shell was used during the simulation. As mentioned above, the traction–separation law was adopted during this simulation, where Maxpe damage (maximum principal strain) of the model needed to be specified. Material properties that were specified included the elastic modulus (E), Poisson's

ratio (ν), density (*ρ*) and maximum principal strain (Maxpe) as shown in Supplementary Table 14. It should be noted that *E* for each phase were approximated based on results of QNM characterisation, and the density was obtained from the physical specimen. The principal strain where a crack forms was set to be 0.0001 which is the tensile strain at which concrete will start cracking [50].

Other parameters that were used during the simulation are summarised in Supplementary Table 15. The model was meshed with quadrilateral elements as presented in Supplementary Fig. 21b, c.

Supplementary Table 14. Material properties of the segmented honeycomb model

Material	Phase	*E* (GPa)	ν	*ρ* (kg/m^3)	Maxpe
Cement	UP	45	0.25	2000	0.0001
	HD	25	0.25	2000	0.0001
	LD	20	0.25	2000	0.0001
	ITZ	15	0.25	2000	0.0001
Polymer	–	3	0.36	1180	0.2

HD, high-density; LD, low-density; UP, unhydrated cement particles

Supplementary Table 15. Parameters used during XFEM simulation

Category	Parameter	Values/option
Damage evolution	Type	Displacement
	Softening	Linear
	Degradation	Maximum
	Mixed mode behaviour	Mode-independent
	Mode mix ratio	Energy
Damage stabilisation cohesive	Viscosity coefficient	1×10^{-5}

A rigid body that acted as the loading plate was placed on top of the model, and it was subjected to downward displacement. The simulation was run in static explicit mode, and results obtained from the simulation included the location of crack initiation and propagation, as well as the stress and strain distribution within segmented honeycomb.

Calculation of material utilisation rate. The material usage of the segmented honeycomb (SH) can be calculated by comparing the volumetric average stress of the SH with that of a model consisting of rectangular-shaped bulk concrete of the same volume. The bulk model takes the load fully in compression, hence the material utilisation rate is 100%. By dividing the average stress of the SH model by the average stress of bulk model, the material utilisation rate (U) can be determined.

$$U(\%) = \frac{\left(\sum_{i=1}^{n} S \cdot w_e\right)_{\mathrm{SH}}}{\left(\sum_{i=1}^{n} S \cdot w_e\right)_{\mathrm{bulk}}} \times 100\% \tag{18}$$

where n is the total number of elements in the model, S is the principal stress of the ith element, and w_e is the volumetric weightage of the ith element calculated as elemental volume divided by total volume of the entire model.

It is important to note that when comparing SH to bulk cement, the strain (i.e. the downward displacement of loading plate) applied on both model needs to be identical. The volume of both the SH and bulk cement models should also be the same. Also, the SH model here is not phase-separated, but assumed to be homogeneous, and has consistent stiffness throughout the model, so the results are comparable to those of the bulk model.

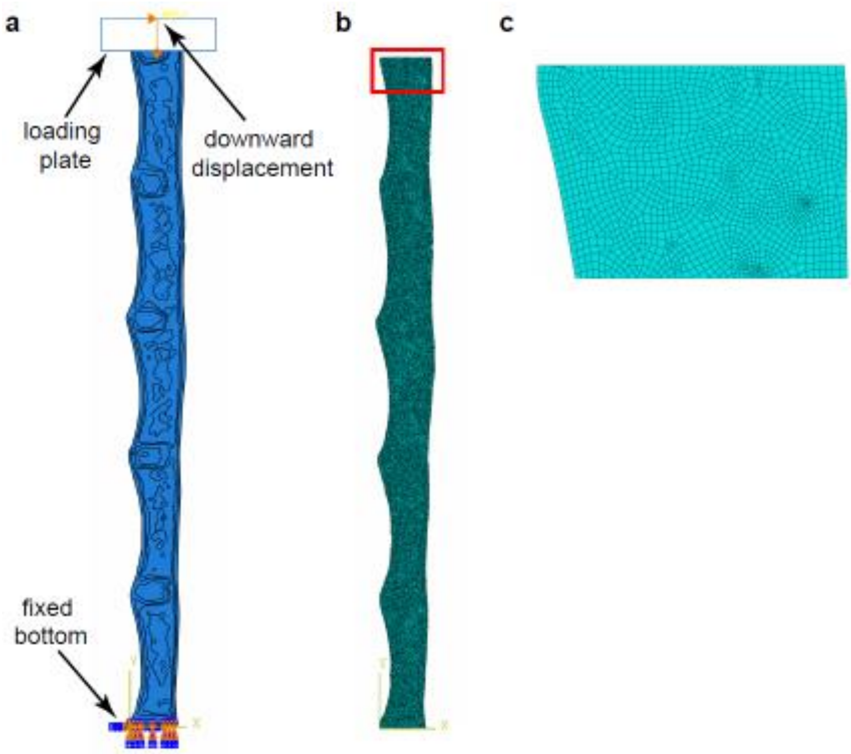

Supplementary Fig. 21. a Modelling of extended finite element method simulation in ABAQUS. **b** Meshed elements of the model. In this case, there are 27 817 nodes and 27 235 elements in the mesh. **c** Magnified view of boxed region in (**b**), showing quadrilateral element within the mesh.

As demonstrated in Fig. 4b, the extended finite element method (XFEM) simulations showed that a lightweight foam structure with randomly distributed pores resulted in large tension regions above and below the pores. As the tensile strength of concrete is significantly lower than its compressive strength, materials in the tensile regions are not utilised for strength [51], which leads to a lower material utilisation rate (59%). In contrast, the tuned SH allows for a better stress flow in the structure due to the continuous wall, which results in a significant reduction of tensile regions (Fig. 4c) and a material utilisation rate of up to 79%.

Material degradation with reduction of density. Efficiency of material usage in lightweight materials is often characterised by the exponential degradation of strength with density [52]:

$$\frac{f_c}{f_{cs}} \propto \left(\frac{\rho}{\rho_s}\right)^n \tag{19}$$

where f_c is the compressive strength, ρ is density and s denotes the respective value of the solid constituent material. $\frac{f_c}{f_{cs}}$ and $\frac{\rho}{\rho_s}$ are defined as relative compressive strength and relative density, respectively.

Supplementary References

1 Hunaiti, Y. M. Strength of composite sections with foamed and lightweight aggregate concrete. *J. Mater. Civ. Eng.* **9**, 58-61 (1997).

2 Weigler, H. & Karl, S. Structural lightweight aggregate concrete with reduced density—lightweight aggregate foamed concrete. *International Journal of Cement Composites and Lightweight Concrete* **2**, 101-104 (1980).

3 Tam, C., Lim, T., Ravindrarajah, R. S. & Lee, S. Relationship between strength and volumetric composition of moist-cured cellular concrete. *Magazine of concrete research* **39**, 12-18 (1987).

4 Kearsley, E. & Wainwright, P. The effect of porosity on the strength of foamed concrete. *Cem. Concr. Res.* **32**, 233-239 (2002).

5 Laukaitis, A., Žurauskas, R. & Kerien, J. The effect of foam polystyrene granules on cement composite properties. *Cem. Concr. Compos.* **27**, 41-47 (2005).

6 Jones, M. R. & McCarthy, A. Preliminary views on the potential of foamed concrete as a structural material. *Magazine of Concrete Research* **57**, 21-31 (2005).

7 Babu, K. G. & Babu, D. S. Behaviour of lightweight expanded polystyrene concrete containing silica fume. *Cem. Concr. Res.* **33**, 755-762 (2003).

8 Haque, M., Al-Khaiat, H. & Kayali, O. Strength and durability of lightweight concrete. *Cem. Concr. Compos.* **26**, 307-314 (2004).

9 Nambiar, E. K. & Ramamurthy, K. Models relating mixture composition to the density and strength of foam concrete using response surface methodology. *Cem. Concr. Compos.* **28**, 752-760 (2006).

10 Yakovlev, G., Kerienė, J., Gailius, A. & Girnienė, I. Cement based foam concrete reinforced by carbon nanotubes. *Materials Science [Medžiagotyra]* **12**, 147-151 (2006).

11 Hiromi, F. & Wee, T. Preparation of high performance foamed concrete from cement, sand and mineral admixtures. *Journal of Wuhan University of Technology-Mater. Sci. Ed.* **22**, 295-298 (2007).

12 Just, A. & Middendorf, B. Microstructure of high-strength foam concrete. *Mater. Charact.* **60**, 741-748 (2009).

13 Nambiar, E. K. & Ramamurthy, K. Influence of filler type on the properties of foam concrete. *Cem. Concr. Compos.* **28**, 475-480 (2006).

14 Nambiar, E. K. & Ramamurthy, K. Shrinkage behavior of foam concrete. *J. Mater. Civ. Eng.* (2009).

15 Mounanga, P., Gbongbon, W., Poullain, P. & Turcry, P. Proportioning and characterization of lightweight concrete mixtures made with rigid polyurethane foam wastes. *Cem. Concr. Compos.* **30**, 806-814 (2008).

16 Nambiar, E. K. & Ramamurthy, K. Sorption characteristics of foam concrete. *Cem. Concr. Res.* **37**, 1341-1347 (2007).

17 Skujans, J., Vulans, A., Iljins, U. & Aboltins, A. Measurements of heat transfer of multi-layered wall construction with foam gypsum. *Appl. Therm. Eng.* **27**, 1219-1224 (2007).

18 Huang, Z., Zhang, T. & Wen, Z. Proportioning and characterization of Portland cement-based ultra-lightweight foam concretes. *Construction and Building Materials* **79**, 390-396 (2015).

19 Tan, X., Chen, W., Hao, Y. & Wang, X. Experimental Study of Ultralight (< 300 kg/m 3) Foamed Concrete. *Advances in Materials Science and Engineering* **2014** (2014).

20 Keertana, B., Mani, S. S. & Thenmozhi, M. Utilization of ecosand and flyash in aerated concrete for a richest mix design. *International Journal of Engineering Science and Technology* **3** (2011).

21 Wegst, U. G., Bai, H., Saiz, E., Tomsia, A. P. & Ritchie, R. O. Bioinspired structural materials. *Nature materials* **14**, 23-36 (2015).

22 *Ceramic Materials Properties Charts*, <https://www.ceramicindustry.com/ceramic-materials-properties-charts/> (

23 *Modulus of elasticity and Poisson's coefficient of typical ceramic materials*, <https://www.sonelastic.com/en/fundamentals/tables-of-materials-properties/ceramics.html> (

24 *Compressive Strength Testing of Plastics*, <http://www.matweb.com/reference/compressivestrength.aspx> (
25 *AZO Materials*, <https://www.azom.com/> (
26 Shafiei, A., Pro, J. W., Martini, R. & Barthelat, F. The very hard and the very soft: Modeling bio-inspired scaled skins using the discrete element method. *J. Mech. Phys. Solids* **146**, 104176 (2021).
27 Ghods, S., Murcia, S., Ossa, E. & Arola, D. Designed for resistance to puncture: the dynamic response of fish scales. *Journal of the mechanical behavior of biomedical materials* **90**, 451-459 (2019).
28 Zhu, D. *et al.* Structure and mechanical performance of a "modern" fish scale. *Adv. Eng. Mater.* **14**, B185-B194 (2012).
29 Martini, R., Balit, Y. & Barthelat, F. A comparative study of bio-inspired protective scales using 3D printing and mechanical testing. *Acta Biomater.* **55**, 360-372 (2017).
30 Vernerey, F. J. & Barthelat, F. On the mechanics of fishscale structures. *IJSS* **47**, 2268-2275 (2010).
31 Zhu, D., Szewciw, L., Vernerey, F. & Barthelat, F. Puncture resistance of the scaled skin from striped bass: Collective mechanisms and inspiration for new flexible armor designs. *Journal of the Mechanical Behavior of Biomedical Materials* **24**, 30-40, doi:https://doi.org/10.1016/j.jmbbm.2013.04.011 (2013).
32 Sutton, G. P. & Burrows, M. Biomechanics of jumping in the flea. *J. Exp. Biol.* **214**, 836-847 (2011).
33 Naleway, S. E., Porter, M. M., McKittrick, J. & Meyers, M. A. Structural design elements in biological materials: application to bioinspiration. *Adv. Mater.* **27**, 5455-5476 (2015).
34 Dalaq, A. S. & Barthelat, F. Manipulating the geometry of architectured beams for maximum toughness and strength. *Materials & Design* **194**, 108889 (2020).
35 Liu, P., Zhu, D., Yao, Y., Wang, J. & Bui, T. Q. Numerical simulation of ballistic impact behavior of bio-inspired scale-like protection system. *Materials & Design* **99**, 201-210, doi:https://doi.org/10.1016/j.matdes.2016.03.040 (2016).
36 Dong, B., Yan, Y. Y. & Li, W. Z. LBM Simulation of Viscous Fingering Phenomenon in Immiscible Displacement of Two Fluids in Porous Media. *Transport in Porous Media* **88**, 293-314, doi:10.1007/s11242-011-9740-y (2011).
37 Anderl, D., Bogner, S., Rauh, C., Rüde, U. & Delgado, A. Free surface lattice Boltzmann with enhanced bubble model. *Computers & Mathematics with Applications* **67**, 331-339, doi:https://doi.org/10.1016/j.camwa.2013.06.007 (2014).
38 Carreau, P. J. Rheological Equations from Molecular Network Theories. *Transactions of the Society of Rheology* **16**, 99-127, doi:10.1122/1.549276 (1972).
39 Filippova, O. & Hänel, D. Grid refinement for lattice-BGK models. *Journal of Computational physics* **147**, 219-228 (1998).
40 Ferraris, C. F., Obla, K. H. & Hill, R. The influence of mineral admixtures on the rheology of cement paste and concrete. *Cement and Concrete Research* **31**, 245-255, doi:https://doi.org/10.1016/S0008-8846(00)00454-3 (2001).
41 Nehdi, M. & Rahman, M. A. Estimating rheological properties of cement pastes using various rheological models for different test geometry, gap and surface friction. *Cement and Concrete Research* **34**, 1993-2007, doi:https://doi.org/10.1016/j.cemconres.2004.02.020 (2004).
42 Johnson, K. L., Kendall, K. & Roberts, a. Surface energy and the contact of elastic solids. *Proceedings of the royal society of London. A. mathematical and physical sciences* **324**, 301-313 (1971).
43 Potyondy, D. O. & Cundall, P. A bonded-particle model for rock. *Int. J. Rock Mech. Min. Sci.* **41**, 1329-1364 (2004).
44 Jing, H., Yin, Q., Yang, S. & Chen, W. Micro-Mesoscopic Creep Damage Evolution and Failure Mechanism of Sandy Mudstone. *Int. J. Geomech.* **21**, 04021010 (2021).
45 Du, M., Chen, S., Duan, W. H., Chen, W. & Jing, H. Role of Multi-Walled Carbon Nanotubes as Shear Reinforcing Nano-pins in Quasi-Brittle Matrices. *ACS Applied Nano Materials* (2018).

46 Wang, W., Chen, S. J., Basquiroto de Souza, F., Wu, B. & Duan, W. H. Exfoliation and dispersion of boron nitride nanosheets to enhance ordinary Portland cement paste. *Nanoscale*, doi:10.1039/C7NR07561H (2018).

47 Chowdhury, I. & Dasgupta, S. P. Computation of Rayleigh damping coefficients for large systems. *The Electronic Journal of Geotechnical Engineering* **8**, 1-11 (2003).

48 Farghaly, A. A. Parametric study on equivalent damping ratio of different composite structural building systems. *Steel and Composite Structures* **14**, 349-365 (2013).

49 Belytschko, T. & Black, T. Elastic crack growth in finite elements with minimal remeshing. *International Journal for Numerical Methods in Engineering* **45**, 601-620, doi:10.1002/(SICI)1097-0207(19990620)45:5<601::AID-NME598>3.0.CO;2-S (1999).

50 Evans, R. H. & Marathe, M. S. Microcracking and stress-strain curves for concrete in tension. *Matériaux et Construction* **1**, 61-64, doi:10.1007/BF02479001 (1968).

51 Richart, F. E., Brandtzaeg, A. & Brown, R. L. A study of the failure of concrete under combined compressive stresses. (University of Illinois at Urbana Champaign, College of Engineering. Engineering Experiment Station., 1928).

52 Gibson, L. J. & Ashby, M. F. *Cellular solids: structure and properties*. (Cambridge university press, 1999).
